# Commentary: “Poverty impedes cognitive function” and “The poor's poor mental power”

**DOI:** 10.3389/fpsyg.2015.01037

**Published:** 2015-07-22

**Authors:** Junhua Dang, Shanshan Xiao, Siegfried Dewitte

**Affiliations:** ^1^Department of Psychology, Lund UniversityLund, Sweden; ^2^Department of Psychology, Stockholm UniversityStockholm, Sweden; ^3^BEE - Behavioral Engineering Research Group, Faculty of Economics and Business, KU LeuvenLeuven, Belgium

**Keywords:** poverty, selectivity of attention, proceduralized processes, self-regulation, distraction, reward sensitivity

Recently, Mani et al. (2013) demonstrated the detrimental effect of poverty on cognitive tasks demanding working memory and logical thinking through a series of experimental and field studies. In the same issue, Vohs (2013) identified these findings as an instance of ego depletion, which led her to predict that the financial strain would entail self-regulatory failures such as overeating and overspending because such strain taxes the poor's limited self-regulatory resources.

Understanding the underlying mechanism and boundary conditions of these remarkable findings is of crucial interest before efficient policy can be built upon them. Mani et al. (2013) attributed their findings to distraction. Due to the limitation of human working memory capacity, the poor tend to fall short of full consideration to other problems as they are preoccupied with pressing budgetary concerns. This suggests a causal, not merely correlational relationship between poverty and mental functions, such that poverty directly impedes cognitive functions because the poor could be easily distracted by monetary concerns (Mani et al., 2013).

Although these authors went through great efforts to safeguard external validity of their independent variable, we contend that they paid insufficient attention to that of the dependent variable. First, the cognitive tasks they used (e.g., IQ tests, the Stroop task) are irrelevant to participants' daily life. It is possible that the poor do not have sufficient motivation to fully engage in these tasks while worrying about their financial situation. Regarding tasks highly relevant to the poor such as financial decision making, the reverse might be found. Indeed, a recent paper by some of the same authors showed that financial concerns increased selectivity of attention, away from irrelevant tasks (which IQ tests arguably are) toward relevant task (which financial decision making arguably is) (Shah et al., 2012). This is also consistent with previous studies showing that stress improves selectivity of attention to task-relevant attributes but reduces utilization of task-irrelevant attributes (Chajut and Algom, 2003). As a result of increased attention, struggling with the financial challenge may lead the poor to gradually get adapted to the enduring financial strain and eventually become proficient and efficient in the domain of financial decision making.

Second, although distraction arising from monetary concerns undermines task performances that rely on working memory, it may not harm cognitive functions (e.g., information-integration category learning) that rely on proceduralized processes running best without heavy demand on working memory and attentional control (Beilock and Carr, 2001; Waldron and Ashby, 2001; Maddox and Ashby, 2004; DeCaro et al., 2011). Instead, distraction has been shown to facilitate such performances since attention is diverted to elsewhere and will not disrupt the learning and execution of proceduralized processes (Markman et al., 2006; DeCaro et al., 2008; Medeiros-Ward et al., 2014). To the extent that the poor are generally engaged in occupations (e.g., driving and typewriting) that require proceduralized processes more often than they require executive controls which rely heavily on working memory, we suggest investigating the impact of poverty induced distraction on tasks relying on proceduralized processes would be equally relevant before policy could build upon Mani et al.'s (2013) findings.

With regard to the influence of poverty on self-regulation, Vohs (2013) argued that the poor tended to enact more self-regulatory problematic behaviors because the financial strain taxes their limited self-regulatory resources. Recently, the limited-resource model has been seriously challenged because: (1) the concept of “resource” is vague and unfalsifiable; (2) more and more empirical findings are hard to reconcile with this model (Kurzban et al., 2013; Inzlicht et al., 2014). Thus, we question the legitimacy of using the limited-resource model to interpret the possible association between poverty and self-regulation failure. Instead, we suggest such association results from two specific mental processes that work in parallel. Self-regulation reflects competition between the force that motivates the impulse and the force that overrides the impulse (Baumeister and Heatherton, 1996). Self-regulation fails when the impulse is relatively strong, when control is relatively weak, or both (Heatherton and Wagner, 2011). From this point, we suggest poverty may lead to self-regulatory collapse by increasing approach-motivated impulses and impeding the control force harnessing impulses.

On the one hand, recent research reveals that eagerness for reward or exposure to a rewarding stimulus can activate a general rewarding system which in turn prompts people to seek anything rewarding (Briers et al., 2006; Van den Bergh et al., 2008; Wadhwa et al., 2008). Thus, such mechanism contributes to the poor's self-regulatory failure as their continued financial deprivation makes them more sensitive to reward cues, which stimulates them to pursue rewards in other domains (e.g., palatable but unhealthy food or expensive goods beyond budget). On the other hand, poverty induced distraction, which was demonstrated by Mani et al. (2013), also contributes to the poor's self-regulatory failure because successful self-regulation is underpinned by basic executive functions including working memory capacity and behavioral control (Hofmann et al., 2012). Compared with seeing the association between poverty and self-regulation through a pessimistic lens such as a continued state of self-regulatory depletion, which is poorly understood and hard to specify or remedy, our proposition elaborates specific processes through which poverty may dampen self-regulation and which may show more fruitful avenues to channel intervention effort.

Further, we suggest poverty does not necessarily lead to self-regulation failure. Previous studies demonstrated that engaging in a *concurrent* inhibitory task (e.g., retrenching expenditure within limited budget) would facilitate self-regulation through an inhibitory spillover mechanism (Tuk et al., 2015) or by blocking individuals from recognizing the tempting value of attractive stimuli (Van Dillen et al., 2013). From this perspective, the poor may excel in self-regulation under certain circumstances. Future studies are needed to specify.

## Conflict of interest statement

The authors declare that the research was conducted in the absence of any commercial or financial relationships that could be construed as a potential conflict of interest.
